# Rabies research in Uganda – A scoping review

**DOI:** 10.1016/j.onehlt.2025.101240

**Published:** 2025-10-14

**Authors:** Rabina Ghimire, Terence Odoch, Samuel George Okech, Rose Lesley Ninsiima, Felister Apio, Siya Aggrey, Felix Opiyo Lakor, Clovice Kankya, Salome Dürr, Sonja Hartnack

**Affiliations:** a Section of Epidemiology, Vetsuisse Faculty, University of Zurich, Zurich, Switzerland; b College of Veterinary Medicine, Animal Resources, and Biosecurity, Makerere University, Kampala, Uganda; c Veterinary Public Health Institute, Vetsuisse Faculty, University of Bern, Bern, Switzerland; d Uganda Wildlife Research and Training Institute, Kasese, Uganda; e Centre for Veterinary Systems Transformation and Sustainability, Clinical Department for Farm Animals and Food System Science, University of Veterinary Medicine Vienna, Vienna, Austria

**Keywords:** Rabies, Uganda, Research, Surveillance, Diagnostics

## Abstract

Rabies remains endemic in Uganda and is a priority zoonotic disease due to its significant public health burden. Although Uganda has committed to eliminating dog-mediated human rabies deaths by 2030, operational and financial challenges persist. A key gap is the lack of a focused research agenda and insufficient funding for its implementation, highlighting the need to map existing research to guide future priorities. This scoping review, thus, aimed at describing and mapping the existing research on rabies in Uganda by identifying, categorizing, and summarizing existing research, while highlighting research gaps to guide future studies and support evidence-based interventions for rabies control and elimination. The scoping review followed PRISMA-ScR guidelines and was registered with the Open Science Framework. A systematic search of PubMed, Web of Science, and Embase databases yielded 110 studies after deduplication. Following screening, 26 original studies regarding rabies research that were based in Uganda were included in the review. Most of these studies were descriptive cross-sectional in design (*n* = 15), including knowledge, attitudes, and practices (KAP) studies (*n* = 5), and surveys and interviews (*n* = 10). Most studies focused on human populations (*n* = 21). Six studies adopted a One Health approach integrating human and animal health sectors. Only two studies employed laboratory methods for molecular characterisation of circulating rabies virus strains, and one study used interventional design to assess vaccination coverage. Notably, no studies implemented consistent and integrated rabies surveillance systems or included economic evaluations of rabies control programs. Our scoping review highlights critical gaps in rabies research in Uganda, particularly the need for intervention-based and longitudinal studies that operationalize the One Health framework. Key areas of research needs include dog vaccination coverage, integrated surveillance systems, diagnostics, and economic evaluations to assess the impact of rabies control and prevention programs. Addressing these gaps is essential for understanding rabies transmission dynamics and strengthening control strategies.

Scoping review registration: https://osf.io/wu5pj

## 1 Background

Rabies is a neglected tropical zoonotic disease causing an estimated 59,000 human deaths per year, with a high economic and health burden, particularly in poor and rural communities of Asia and Africa [1]. Worldwide, over 99 % of human rabies deaths are due to bites from infected dogs [2]. The disease is almost 100 % fatal when clinical signs appear [2,3], but it is entirely preventable with timely administration of vaccines, known as post-exposure prophylaxis (PEP), following an exposure through scratches or bite of infected animals [4]. Eliminating dog-mediated rabies is only possible by tackling it at the reservoir population, primarily dogs, through vaccination [1,2]. Until this is reached, rabies continues to cause numerous human deaths.

Rabies, being a zoonotic disease, requires One Health approach for its effective control, prevention, and eventual elimination. Recognizing the interconnection between human, animal and environmental health, the quadripartite, comprising of the World Health Organization (WHO), the World Organization for Animal Health (WOAH), the Food and Agriculture Organization of the United Nations (FAO), and United Nations Environment Programme (UNEP) through their advisory panel One Health High-Expert Panel (OHHLEP) has defined One Health as “an integrated, unifying approach that aims to sustainably balance and optimize the health of people, animals and ecosystems. It recognizes the health of humans, domestic and wild animals, plants and the wider environment (including ecosystems) are closely linked and interdependent” [5]. Building on this, the One Health Joint Plan of Action (OHJPA) was developed to foster collaboration across these three sectors with an aim of enhancing disease prevention, surveillance, and control measures. Within this, the Theory of Change (ToC) Action framework outlines three strategic pathways to achieve outcomes and impacts by systematically planning and evaluating the interventions [6]. Specifically, Pathway 3 focuses on strengthening the scientific evidence base, translating knowledge into actionable data, and improving technical tools, protocols, guidelines, and surveillance systems [6].

Uganda is an East African country endemic for rabies where nearly 90 % of the population reside in communities at risk for canine rabies transmission, with an estimated 36 human deaths annually and a reported bite incidence of 25 bites/100000 population [[7], [8], [9]]. Although these figures may represent underestimation due to underreporting and surveillance limitations [7], rabies is one of the prioritised zoonotic diseases [10]. Recognizing the need to streamline rabies control efforts and align with global elimination goals, Uganda validated a National Rabies Elimination Strategy (NRES) in 2022, with support from the World Organization for Animal Health (WOAH) [11]. Also, Uganda has committed to the global Zero by 30 initiative but remains in the early stages of rabies control efforts. “Zero by 30” is a joint goal set by the WHO, FAO, WOAH and the Global Alliance for Rabies Control (GARC) to eliminate dog-mediated human rabies deaths by 2030 [12]. Uganda faces significant operational and financial challenges, particularly with efforts such as mass dog vaccination and PEP, that concentrated in urban areas, while rural regions—where the rabies burden is often higher—receive limited coverage due to financial and human resource constraints. In the Stepwise Approach for Rabies Elimination (SARE) assessment conducted in 2017, the country scored 0.5 out of 5, indicating that situational data were being collected and analysed to design local-level interventions [13].

To strengthen intersectoral collaboration for rabies management, a workshop was organised in March 2024 in partnership with the German Epidemic Preparedness Team (SEEG), Deutsche Gesellschaft für Internationale Zusammenarbeit (GIZ), Germany. This workshop facilitated a comprehensive SWOT (Strengths, Weaknesses, Opportunities, and Threats) analysis to evaluate Uganda's National Rabies Elimination Strategy (NRES) across four thematic areas. A key weakness identified under the theme of Training and Operational Research was the absence of a focused rabies research agenda and the lack of sufficient advocacy for research funding. While some research has been conducted on rabies in Uganda, significant gaps remain, as highlighted in the SWOT analysis. It is important to analyse the types of research conducted, identify key findings, and outline the necessary next steps for rabies control.

Therefore, this scoping review aims to describe the research conducted on rabies in Uganda by identifying, mapping, categorizing, and summarizing existing studies. This will help to identify the gaps in the current knowledge base and determine areas for future research, guiding further studies and supporting evidence-based interventions for rabies control and elimination.

## 2 Methods

### 2.1 Protocol design and registration

This scoping review was conducted according to the Preferred Reporting Items for Systematic Reviews and Meta-Analyses extension for scoping review (PRSIMA-ScR) guidelines, aligning with the framework of O'Malley, Arksey, and Joanna Briggs [14], further developed by Tricco et al. [15]. The final protocol was registered with the Open Science Framework (OSF) [16].

The stages of the study were comprised of the following steps: i) Identification of the research questions and objectives, ii) identification of relevant published studies, iii) selection of studies and documents, iv) extracting and charting the evidence and data, and v) collating and summarizing the results and identifying research gaps on rabies research in Uganda.

A multi-disciplinary team, involving veterinarians, epidemiologists, public health specialists, and environmental scientists jointly looked over the application of the protocol, inclusion, and exclusion criteria as well as the review process. This diverse expertise was included to ensure that the scoping review incorporates various perspectives and addresses all relevant aspects, improving the quality and comprehensiveness of the findings.

The scoping review was guided by the following research questions:

1) What is the nature of authors' involvement in rabies research conducted in Uganda?
  a. Were the authors affiliated with Uganda?
  b. What was the extent of international collaboration?
2) What research methods and study designs have been employed in rabies research in Uganda?
  a. Were the studies quantitative, qualitative, or mixed methods?
  b. Which type of study design was used (for example, case series, cross-sectional, cohort, case-control, key informant interviews, focus group discussions)?
  c. What was the target population?
  d. What sampling methods were used? Was the sample size predetermined?
3) What interventions, outcomes, and risk factors were assessed in the studies?
4) What was the extent of data information and availability in the studies?
  a. Were data and information openly available?
  b. Were FAIR (Findable, Accessible, Interoperable, and Reusable) data principles applied?
  c. Were the studies pre-registered?
  d. Was funding information available?
  e. Was ethical approval reported?
5) Was the research conducted in a One Health framework?
  a. Was there an intersectoral collaboration between the human and the animal health sectors?
  b. How have environmental factors and the ecosystem been considered?
  c. Can the type of research be considered transdisciplinary (i.e., including communities or other non-academic actors besides the researchers)?
  d. Was the added value of applying a One Health framework assessed?
6) What type of surveillance systems, including diagnostics, have been considered?
  a. Was there any link to electronic (e.g., Integrated Disease Surveillance and Response (IDSR), District Health Information Software (DHIS2), or others) or paper-based approaches for surveillance?
  b. Were diagnostic methods used in the studies? Was information on diagnostic tests given?
7) Was there any economic assessment of rabies control performed? If yes, how was it performed?
8) What were the challenges and gaps in rabies research identified in the studies?

### 2.2 Eligibility criteria

Studies were included in this review if they explicitly focused on rabies or had a clear connection to the disease, were based in Uganda either exclusively or as part of a broader study and constituted original research. In contrast, reviews and opinion papers were excluded from the analysis. However, these studies were still screened and investigated to identify any relevant references that met the inclusion criteria and could provide valuable insights to the review.

### 2.3 Information sources

Potentially relevant studies were searched across three electronic databases with scientific literature: PubMed**,** Web of Science, and Embase**.** No language restrictions were applied to the search, ensuring inclusivity of studies in various languages. No date restrictions were applied. The review included all publications up to October 2024.

The search strategy was performed by a professional librarian at the University of Zurich (Supplementary File 1). After deduplication in Endnote, the retrieved literature was imported into Rayyan software [17] for further screening.

### 2.4 Screening and selection of studies

In Rayyan, a two-stage selection process was performed. In the first step, titles and abstracts were screened in Rayyan pertaining to the inclusion criteria by at least two reviewers (RG, SH, TO, SGO, SD, SA, RN, FA, FO) for each study in a blinded manner. In case of disagreement between reviewers, consensus was attained through discussion or third reviewer. In the second step, the studies included from this screening were checked for full text following the same process. From this, the final corpus of the review for data extraction was selected.

### 2.5 Extracting and charting the evidence and data

Data charting was performed in Microsoft Excel after the identification of relevant studies for the review. Each study was extracted by at least two reviewers (RG, SH, TO, SGO, SD, SA, RN, FA, FO). These data were then collated, compared, and discussed.

Data chart included the following details pertaining to the research questions:

• **Publication details:** Citation details (e.g., doi), year of publication, authors, authors affiliation with geographic details
• **Study details:** Aim/objectives of the study, study design**,** research types (quantitative, qualitative), study design details (e.g., observational, interventional, interviews)**,** target population, interventions assessed**,** primary outcome**,** secondary outcome(s)**,** risk factors assessed**,** application of One Health framework**,** surveillance systems, diagnostic tests and sampling**,** economic assessment**,** integration with other animal diseases, data accessibility, application of FAIR principles, ethical approval, pre-registration, funding, research gaps and challenges stated in the study

### 2.6 Data analysis

The current state of rabies research in Uganda was synthesized using key findings and themes across the literature. Data was visualized using R *(Version 2024.04.2 Build 764)*. The DiagrammeR [18] package was used to create the flowchart.

## 3 Results

### 3.1 General findings

**Fig. 1:**
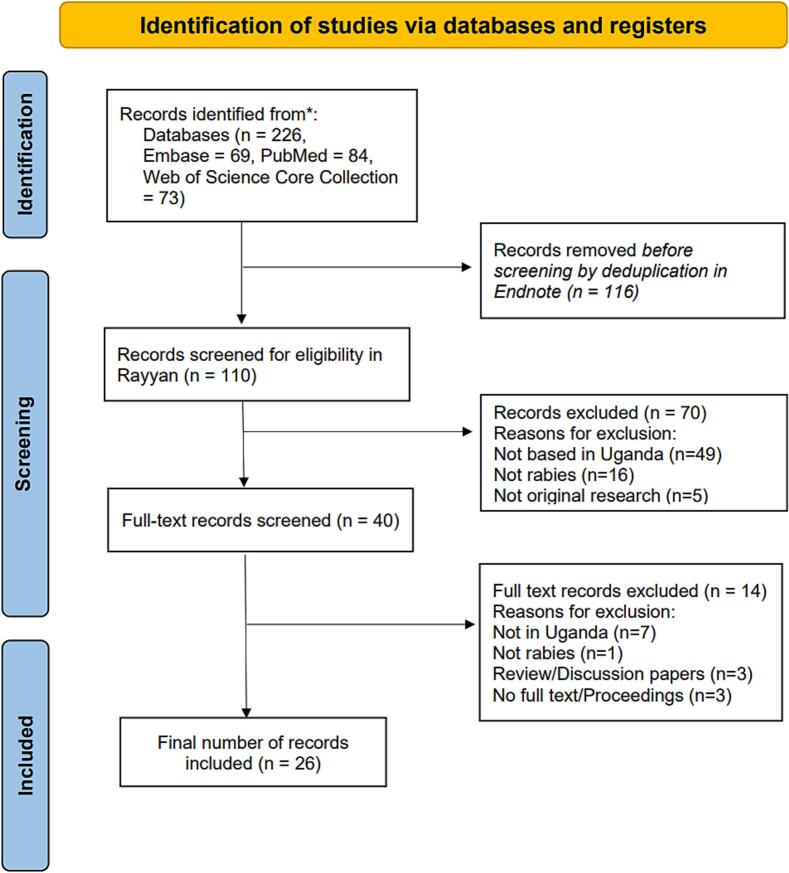
PRISMA flow diagram showing the number of studies retrieved, screened, and included on rabies-related research in Uganda.

The PRISMA flow diagram depicts the number of records retrieved, screened and included in the analysis as shown in Fig. 1. In the literature search, we identified a total of 225 records. Following deduplication, 110 records were screened for titles and abstracts. Out of 40 studies selected for full-text review, 26 studies were included as a final review corpus (Supplementary File 2).

**Fig. 2:**
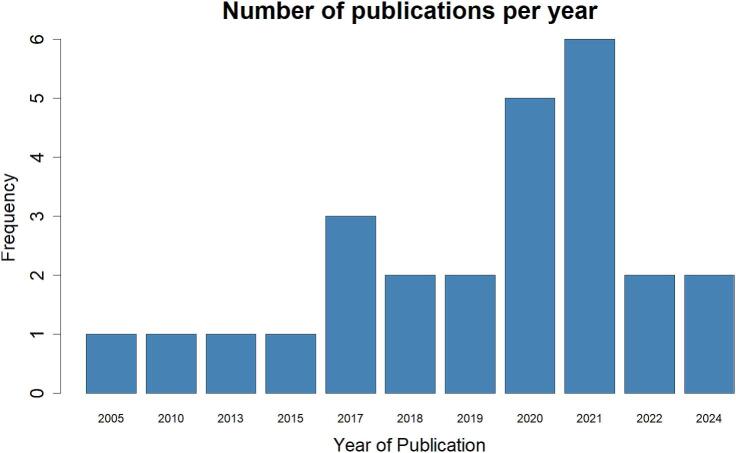
Number of rabies-related publications by year in Uganda.

The publication year ranged from 2005 to 2024, with the maximum papers published after 2017 (Fig. 2). A noticeable peak was around 2021, where the number of publications reached 6, indicating a significant increase.

#### 3.1.1 Geographic focus

While the majority of studies (*n* = 21) focused exclusively on Uganda, five studies included Uganda as a part of a wider study in rabies or zoonotic diseases [[19], [20], [21], [22], [23]].

#### 3.1.2 Author's involvement and international collaborations in rabies research

Among the identified studies, 22 involved Ugandan researchers based on their affiliation, with five conducted exclusively by Ugandan researchers [9,[24], [25], [26], [27]]. In contrast, four studies [[19], [20], [21],^28^] had no involvement of any Ugandan researchers. These studies were generally a part of broader research conducted across Eastern or Sub-Saharan Africa, except for the study by Hirano et al., 2010 [28], which solely focused on Uganda.

Rabies research in Uganda involved numerous international collaborations. The United States of America (USA) had the highest number of rabies research collaborations (*n* = 8) [7,10,[29], [30], [31], [32], [33], [34]], followed by Kenya (*n* = 5) [20,[30], [31], [32], [33]], the United Kingdom (n = 5) [[21], [22], [23],^35^,^36^], Switzerland (*n* = 4) [22,23,37,38], and South Africa [21,30], Chad [22,23], France [34,39], Guatemala [22,23], Indonesia [22,23], and Norway [40,41] each contributed two studies. Additionally, Ethiopia [19], Andorra [39], Ghana [20], Italy [20], Japan [28], Spain [39], and the Netherlands [19] each had one publication related to rabies research in Uganda.

### 3.2 Research methods and study design

**Fig. 3:**
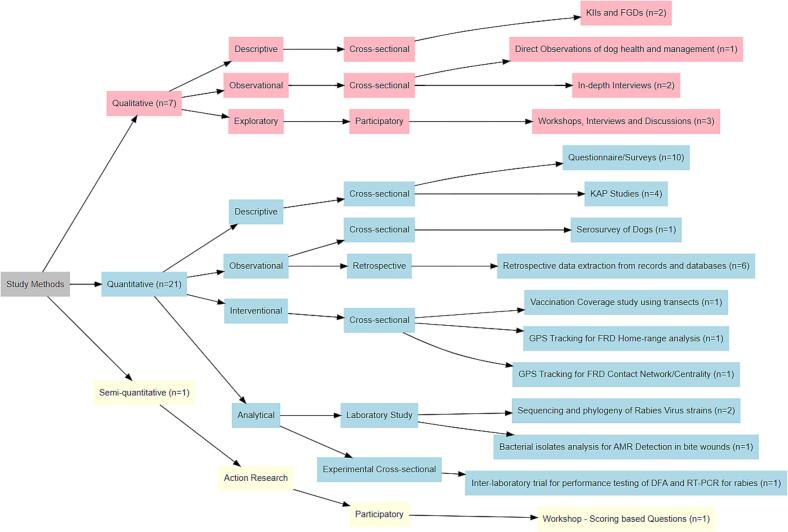
Flowchart illustrating the classification of study methods, study designs, and approaches with corresponding frequencies (number (n)). Colours are only used for visual distinction. The total n does not correspond exactly to 26 because some studies employed more than one study method and design.

Fig. 3 demonstrates the study methods and designs with their corresponding frequencies. The studies employed qualitative (*n* = 3) [21,37,38], quantitative (*n* = 20) [7,9,19,20,[22], [23], [24], [25], [26], [27], [28], [29],[32], [33], [34], [35], [36],[39], [40], [41]], mixed-method (n = 3) [27,30,31], and semi-quantitative approaches [10] to collect data. Qualitative methods included KAP studies using key informant interviews (KIIs) and focus group discussions (FGDs) [27,38], or KIIs and FGDs for policy mapping [21], direct observations of dog health, care and management [38], and in-depth interviews with rabies bite victims, healthcare workers and veterinarians [30,31], and participatory exploratory methods using workshop and interviews [37].

For quantitative studies, descriptive cross-sectional studies were the most common, with KAP studies (*n* = 5) [7,27,29,34,40], and questionnaire interviews and surveys (*n* = 10) [7,20,22,23,26,[31], [32], [33],^36^,^41^] being the most frequently used designs. Additionally, an observational serosurvey of dogs was conducted to assess rabies antibody prevalence [39]. Studies also used observational retrospective data from previous health records and databases. Interventional studies (*n* = 3) used transects for vaccination coverage estimation for static point mass dog vaccination [35] and GPS tracking in FRDs for home range analysis [22] or centrality/contact network of free-roaming dogs (FRDs) [23]. Analytical studies (n = 3) included laboratory methods like sequencing and genetic characterisation of rabies virus strains [28,34], to check for double risk of rabies and presence of antimicrobial resistant microorganisms in wounds of bite victims [32], and interlaboratory trial for diagnostic test performance of Direct Florescence Antibody Test (DFAT) and Reverse Transcriptase Polymerase Chain Reaction (RT-PCR) for rabies virus [20].

One study employed semi-quantitative methods, using a participatory approach through a workshop with scoring-based questions for a zoonotic disease prioritisation exercise [10].

### 3.3 Target population

Table 1 highlights the number of studies categorised by target populations. Most studies predominantly focused on human populations (*n* = 21), while fewer addressed animal populations (*n* = 9) or both populations (*n* = 4) [22,23,34,35]. Most studies on human populations targeted households, community members, and dog owners, as well as dog bite victims. Key professional groups include veterinary professionals [27,31,37,38], physicians and healthcare workers [27,30,31,37,38], and community health workers, leaders, school teachers and school children [38], or policy experts [10,21]. Among animal focused studies, dogs were the target (*n* = 8) [9,19,22,23,28,34,35,39], with some studies addressing livestock (*n* = 3) [24,28,34] or wildlife populations [34].

**Table 1:** Studies according to their target populations with corresponding frequencies (number (n)).

Domain of target Population	Frequency (n)	Target population classification	Frequency (n)
Humans	21	Veterinarians/Veterinary professionals [27,31,37,38]	4
		Physicians/health care workers [27,30,31,37,38]	5
		Community workers, Village health team members, local extension workers (human and animal health) [38]⁎	1
		Households, community members and dog owners [7,22,23,29,34,35,38,40,41]	9
		Dog bite victims/medical records of human dog bite victims/cases of human bite victims [9,25,26,[29], [30], [31], [32], [33],^36^]	9
		School teachers and school children [38]	1
		Experts/policy makers [10,21,37]	3
Animals	9	Dogs [9,19,22,23,28,34,35,39]⁎	8
		Livestock [24,28,34]⁎ (Cattle, Goats, Sheep)	3
		Wildlife (fox, jackals) [34]⁎	1
Laboratories	1	Rabies laboratories in West, Central and East Africa (sub-Saharan) [20]	1

⁎ Studies that included more than one target population (from human and animal sector). The total *n* does not match the total number of 26 studies included because some studies focused on multiple population groups.

### 3.4 Sampling methods

Various sampling methods were used across the studies included in the review. Convenience and purposive sampling were most commonly used (*n* = 10) [10,20,22,23,[30], [31], [32], [33],^38^,^41^], while snowball sampling was used by one study [21]. Multistage cluster sampling was utilised by two studies [29,40]. Random selection of participants [27] or households [22,23], or randomly stratified sampling based on bite reporting system and geographical area [7] were conducted. In two instances, data were collected from a consecutive series of patients [26,36], where a district was purposively [26] or randomly chosen [36]. Three studies conducting retrospective trend analysis included all recorded animal bites [9,25] and human deaths [25]. One study included all accessible dog population in the area for vaccination [35].

Five studies predetermined sample size [7,27,29,40,41], where one [7] didn't reach the expected sample size. In seven studies [19,20,24,28,35,42,43], sample size predetermination was not applicable.

### 3.5 Interventions, outcomes and risk factors assessed

The studies included in this scoping review assessed a variety of rabies interventions, categorised into key themes with primary and secondary outcomes, and assessed risk factors (Table 2).

**Table 2:** Rabies interventions assessed in the studies with their respective outcomes and risk factors with their corresponding frequencies (number (n)).

Interventions	Study	Primary outcomes	Secondary outcomes	Risk-factors assessed
Dog vaccination (*n* = 7)	[7,35]⁎	Vaccination coverage, human-dog ratio	Demographic characteristics, reasons / attitudes towards vaccination Dog population size [7]	Age (Puppy vs adult), Socio-economic status of owners [35] Household demographics, economic level, animal care [7]
	[9]⁎	Retrospectively assessed pet vaccination coverage	–	region, year
	[22]⁎	Vaccinated dogs	–	Dog's age, sex, BCS, role (guardian dog/Shepard dog/hunting dog), free roaming time, study site
	[7,27,34,41]⁎	Knowledge and practice of dog vaccination	–	Distance to park, sociodemographic and practice (hunting, fetching wood and water) [41]
Dog management, Behaviour and ownership (*n* = 4)	[38] ⁎	KAP of community members towards dog health for rabies and dog management practices	–	–
	[7]⁎	KAP on dog ownership		Poverty level
	[22] ⁎	Dog home range	Management practices (feeding, confinement, transportation)	Dog's age, sex, BCS, role (guardian dog/Shepard dog/hunting dog), free roaming time, study site
	[23]	Dog contact networks (structure of FRDD contact network (centrality))	Factors shaping the structure of FRDD contact network (centrality)	Demographic factors of dogs and owners
Human bite case management (Treatment, wound washing, PEP) (*n* = 6)	[29,34,36]⁎	Health seeking behaviour of dog bite victims (treatment, wound washing, and PEP)	–	Knowledge of rabies, bite, and wealth scoring [29] Age, gender, dog ownership, care and number of bites [34]
	[33,36]	Time elapsed until PEP after dog bite	–	Socio-demographics and clinical information
	[31]	Factors of compliance with treatment guidelines for dog bite victims	–	Factors (socio-demographic, bite event, circumstances) influencing response to dog bites
	[30]	Assessment of adherence to Uganda clinical rabies guidelines	Challenges in the clinical management of dog bites	–
Rabies transmission (n = 3)	[27,34,40]⁎	KAP on rabies and it's transmission Interaction between dog-wildlife for rabies transmission [40]	Dog-wildlife-livestock contact risk practices [40]	District, education level [40]
Data surveillance and reporting (n = 7)	[19]	Reported dog rabies cases to multinational organisations	–	Year (trend of time)
	[25]	Retrospectively assessed animal bites, human rabies death	Geographical and temporal cases	Age, sex, region
	[9]⁎	Retrospectively assessed animal bites, human PEP administration, dog samples tested for rabies	Development over time	Region, year
	[36]⁎	Incidence of animal bite injuries	Rabies mortality from bite injuries	Demographic and clinical information
	[26]	Animal bites injuries recorded	Characteristics of the dogs (and others)	Socio-demographic characteristics of the patient, clinical characteristics of the bite and treatment
	[41]	Incidence of rabies (humans, dogs, and livestock)	Mortality due to rabies	Distance to national park, sociodemographic, and practice (hunting, fetching wood and water)
	[24]	Retrospectively diagnosed cases of cattle diseases, including rabies	–	Monthly precipitation
Laboratory (n = 5)	[20]	Diagnostic test accuracy (proficiency testing: sensitivity, specificity, kappa, concordance) (DFAT, PCR)	Questionnaire responses	Time elapsed before results were sent
	[34] ⁎	Lab test results, sequencing and genetic characterisation	–	–
	[28]	Rabies virus sequence and phylogeny	–	–
	[32]	Bacteriology, susceptibility, management of dog bite wounds	Sociodemographic, wound management including some knowledge & attitude on dog bites and rabies	
	[39]	Serum antibody titres for rabies	Titres to other canine infections	National Park
Culling (n = 1)	[37]	Evaluate Ropohl's responsibility checklist to reduce moral distress	Empower understanding and advocacy in individuals	Culling dogs through poisoning posses' risks of poisoning other animals including humans
Policy making for rabies control initiatives (*n* = 2)	[21]	i) Connections or links between global and African zoonotic disease policies, ii) Key stakeholders in Nigeria/Uganda for policy making for public health and their interconnectedness, iii) Political context of zoonotic disease control in Uganda/Nigeria	–	–
	[10]	Prioritisation list of zoonotic diseases, including rabies	Action plan for multisectoral implementation	–

The total *n* does not match the total number of 26 studies included because some studies focused on multiple interventions.

In this context, the term ‘intervention’ is not used in the classical experimental sense, but rather refers to any action, strategy, or practice aimed at rabies prevention and control. While some studies directly implemented such interventions, others evaluated or documented them through observational methodologies, including KAP surveys, questionnaires, KIIs, FGDs, and other qualitative or quantitative approaches.

⁎ Studies assessed more than one intervention.

Seven studies reported dog vaccination [7,9,22,27,34,35,41], focusing on vaccination coverage, numbers vaccinated, and KAP related to vaccination.

Dog management, behaviour, and ownership included four studies [7,22,23,38], primarily investigating the KAP of community members towards dog management practices, dog home ranges and contact networks, and behaviours like feeding and confinement.

Six studies explored the theme of human bite case management [[29], [30], [31],^33^,^34^,^36^], examining responses of dog bite victims to seek treatment and PEP, healthcare workers' compliance with guidelines, and challenges in bite case management.

Three studies assessed KAP on rabies symptoms and transmission patterns [27,34,40], including KAP transmission dynamics and wildlife-domestic interactions [40].

Data surveillance and reporting were addressed in seven studies [9,19,[24], [25], [26],^36^,^41^], covering reported or retrospectively assessed human or dog rabies cases, animal bites or PEP administration.

Five studies that reported laboratory analysis [20,28,32,34,39], focused on virus characterisation and sequencing, wound management, diagnostic methods proficiency testing and serum antibody titres.

One study addressed ethical implications of dog culling [37], while two studies [10,21] focused on policy making process for rabies control, stakeholder involvement and cross-regional collaborations.

### 3.6 Data and information availability

Data were available in the manuscript or supplementary file on five studies [19,20,23,29,43], while one study deposited the data in GenBank [28]. In 12 studies, authors indicated that data could be shared upon request [7,22,25,27,[30], [31], [32], [33], [34], [35],^38^,^40^,^41^].

The application of FAIR principles was found across four studies [19,23,29,43], whereas in the remaining 22, FAIR principles were not applied.

Most studies (*n* = 25) provided the funding information. Among these, 24 studies reported receiving funding for the studies, while one mentioned the absence of funding support [24].

None of the studies were pre-registered.

### 3.7 Ethical approval

Eighteen studies acquired ethical approval from various national and international institutions like, Makerere University School of Public Health Research Ethics Committee (SPHREC) [40,41], Makerere University College of Veterinary Medicine, Animal Resources, and Biosecurity (COVAB) [27], Makerere University School of Biosecurity, Biotechnical and Laboratory Sciences Research Ethics Committee [38], Centre for Disease Control and Uganda Virus Research Institute [29], the University of Edinburgh [35], Committee on the Ethics of Animal Experiments of the IZSVe, Italian Ministry of Health [20], Uganda National Council for Science and Technology (UNCST) [22,23,25,[30], [31], [32], [33]], International Coordinating Partner Countries (ICPCs) [21], Ethics committee of One Health (Ministry of Agriculture) [34], Centre for Disease Control and Prevention's Human Subjects Research Office [7], and Mulago Hospital's Research and Ethics Committee [26]. Ethical approval was not required for some studies, such as retrospective analyses of registered human or animal rabies cases, due to the nature of the research.

### 3.8 Intersectoral collaboration, one health approach, and transdisciplinarity

Among the 26 studies reviewed, 12 demonstrated intersectoral collaboration in rabies research, although this was largely limited to research authors' collaboration from human and animal health sectors [31,36,40]. A few studies additionally included collaborations from women and gender studies [41] and government ministries, including the Ministry of Health (MoH) and the Ministry of Agriculture, Animal Industry and Fisheries (MAAIF) [9,25]. A few studies reflected a One Health approach integrating data from human and animal health professional informants [27,31,38,40], with two studies expanding their scope to agriculture [21], and wildlife, water and environment [10]. Two studies [40,41] highlight the increased risk of rabies for households near national parks as well as focusing on KAP in communities near these areas. A study combined KAP surveys from households with animal rabies virus characterisation [34], while another study evaluated vaccination surveys and surveyed dog owners for human canine demography [35]. Two studies also focused on GPS tracking of dogs alongside household questionnaires [22,23].

Considering the environment and ecosystem, four studies assessed the closeness to game reserves or national parks [35,[39], [40], [41]], while two studies incorporated the ecosystem partly by including the aspects of climatic variations (rainy/dry seasons) [22,24], or comparing rural and urban settings [22,23]. Three studies reported engaging local communities [21,22,38], school children and their parents [38], or government, private sectors, and research institutes [21].

### 3.9 Integration of animal, human or wildlife diseases

Three studies in this review explored the integration of animal, human or wildlife diseases. A study conducted a serosurvey of dogs to assess exposure to pathogens affecting human, livestock, and wildlife health, including Canine Distemper Virus, Canine Parvovirus, *Leptospira interrogans*, Leishmaniasis, *Toxoplasma gondii*, and *Neospora caninum* [39]. From a policy perspective, one study examined systemic challenges in controlling endemic and neglected zoonotic diseases [21]. A 2017 multisectoral initiative in Uganda identified 48 zoonotic diseases, prioritising seven - Anthrax, Zoonotic Influenza, Viral haemorrhagic fevers (Ebola, Marburg, CCHF and RVF), brucellosis, African trypanosomiasis, plague, rabies [10].

### 3.10 Link to rabies surveillance systems

Seven studies indicated some linkage to the surveillance systems [9,19,24,25,[34], [35], [36]]. Specifically, one study mentioned the use of international databases for surveillance purposes through reporting dog bites in-country [19], while a study noted the use of Integrated Disease Surveillance and Response (IDSR) for passive surveillance [36]. A retrospective analysis of registries in the national reporting database [9,24,25] as well as health centres [34] was conducted to assess rabies cases. A study reported the use of the “Mission Rabies” app during vaccination campaigns, contributing to surveillance efforts [35].

### 3.11 Diagnostics

Three studies utilised laboratory diagnostics for rabies detection and characterisation. One study performed an indirect fluorescent antibody test (iFAT) and Reverse Transcriptase Polymerase Chain Reaction (RT-PCR) to detect rabies virus in already existing samples and characterise the circulating rabies virus strains [34]. Similarly, a study focused solely on molecular characterisation of the virus using RT-PCR that had already tested positive by iFAT [28]. A seroprevalence study was conducted to determine antibody titres against the rabies virus, using the Fluorescent Antibody Virus Neutralization (FAVN) test [39].

### 3.12 Economic assessment

In one study, although an economic assessment was not conducted, social and economic aspects were included among five criteria for multisectoral zoonotic disease prioritisation [10]. None of the other studies included an economic assessment or analysis.

### 3.13 Limitations, challenges and gaps identified in the reviewed studies

This review highlights several limitations and challenges that were reported by the included studies on rabies research in Uganda. Many studies noted data generalizability as a challenge, because studies often focused on households with dogs or livestock, excluding broader populations [41]. Additionally, unowned dogs in uninhabited areas were underrepresented, limiting the scope of findings as cited [35]. Many studies relied on self-reported data, which they acknowledged as being prone to recall or information bias, inaccuracies, and variability in respondents' knowledge of rabies symptoms [22,29,31,33,41]. These biases were compounded using cross-sectional designs, which restricted causal-effect assessments and led to potential misclassifications [29,33]. Short observation periods and incomplete data collection were also noted as barriers to drawing robust conclusions [22,23].

Surveillance limitations were also frequently mentioned, with passive systems affected by underreporting, inconsistent delivery of HMIS reports to the Ministry of Health and missing key data such as case locations or details of biting animals [25]. Wide age categories in surveillance data limited analysis of age-specific risk factors, and PEP outcomes could not be reliably linked to rabies deaths due to the lack of laboratory-confirmed cases and human/dog population data [9]. Methodological gaps were reported, including the absence of logistic regression analyses, FGDs, and KIIs, along with desirability bias in self-reported practices, which impacted data quality and interpretability [40].

## 4 Discussion

Uganda has committed to achieving the “Zero by 30” goal to eliminate dog-mediated human rabies deaths by 2030. Uganda has a rabies control plan, guided by the NRES (2022−2030). However, the current state of research supporting this goal has not been previously assessed. This scoping review presents the first comprehensive review of rabies research in Uganda from 26 selected studies, identifying the areas of focus of research, research trends, and gaps identified in the existing literature. Furthermore, this scoping review aligns with pathway 3 of this ToC framework for strengthening the scientific evidence base, translating knowledge into data, and improving technical tools, protocols, and guidelines [6].

The increase in rabies research in the past few years highlights the growing attention to rabies in Uganda. This increase aligns with the establishment of the One Health Platform in 2016 and implementation of the Uganda One Health Strategic Plan 2018–2022 [44]. A multisectoral approach prioritised zoonotic diseases, with rabies recognised among the seven prioritised diseases [10], though competing priorities such as tuberculosis or malaria, FMD, often limit the funding and resources. In alignment with the global goal to eliminate dog-mediated human rabies deaths by 2030, efforts have led to the drafting and validation of the NRES to support the global *Zero by 30* goal [11]. However, the limited research conducted so far, as demonstrated by the inclusion of only 26 studies in this review, highlights the need for further research to advance rabies elimination and prevention efforts.

While the majority of studies involved Ugandan researchers, some research was conducted in Uganda without the local participation of researchers [21,28]. On the other hand, most research collaborations originated from and were funded by the Global North, with five studies [9,[24], [25], [26], [27]] conducted exclusively by Ugandan researchers. Greater engagement of local experts is essential to ensuring research aligns with national needs and priorities, such that studies are both impactful and reflective of the local context. Increased participation of Ugandan researchers would enhance capacity building, inclusion, and leadership to produce studies that are both impactful and representative of the local context. Therefore, there is a need for increased in-country motivation, investment, and institutional responsibility to drive and lead research efforts within Uganda.

The majority of the studies consisted of descriptive research, predominantly focusing on KAP studies, surveys and questionnaires. Additionally, some studies employed observational and qualitative assessment methods. This trend aligns with findings from other scoping and systematic reviews across Africa, where a similar study design dominates the rabies research landscape [45,46]. While these studies provide valuable insights into the level of rabies awareness, behavioural trends, and support evidence-based interventions, a critical limitation is the lack of longitudinal interventional studies. This gap is particularly concerning for rabies surveillance, control and prevention strategies, as it limits the translation of knowledge into field-based integration and tangible public health outcomes. Given that mass dog vaccination is the primary prevention and control measure for rabies, studies assessing vaccination coverage should be a priority. However, only one study has evaluated this crucial aspect [35], highlighting a significant gap in research. Furthermore, aside from studies focused on understanding the KAP of bite victims regarding rabies vaccination, there is a significant lack of educational interventional studies aimed at improving rabies prevention, particularly within communities and among children. In this regard, previous research in Sri Lanka and the Philippines highlights the importance of these studies for raising awareness and strengthening rabies prevention through promotion of responsible ownership and timely healthcare seeking, especially among the vulnerable population in the community, such as children [47,48]. Additionally, we observed significant research gaps in vaccine production, efficacy assessment, and post-vaccination antibody titres in the Ugandan context. Previous research undertaken by Neevel et al. 2018 to prioritise rabies research agendas in endemic countries also emphasises a deeper understanding of the factors that hinder the effectiveness of mass dog vaccination programs and the development of affordable PEP for humans [49]. Thus, strengthening research efforts beyond descriptive studies by incorporating intervention-based and longitudinal studies would be instrumental in driving sustainable rabies control strategies.

Although Uganda has embraced One Health principles with the One Health platform and the One Health strategic plan, only a few studies explicitly integrate human, animal, and environmental health data. Two studies that extended beyond human and animal health to include agriculture, wildlife, water, and the environment were policy-focused, aimed at neglected zoonotic disease control or multisectoral prioritisation [10,21]. The limited number of studies shows that there remains a gap in operationalizing the One Health frameworks into interventions, rather than limiting them to policy discussions. One study in this review emphasised the added value of One Health collaboration in rabies research [27]. However, the current research landscape in Uganda lacks applied studies that demonstrate the effective implementation of this approach. Broader national insights also point to progress in applying the One Health framework, with reports indicating strengthened early detection, reporting, and response to zoonotic diseases such as rabies [43]. Also, research on rabies transmission at the wildlife-livestock-human interface remains limited, with one study in this review addressing the issue, despite the critical role wildlife may play in disease spillover. Understanding these transmission dynamics is essential for developing a comprehensive rabies elimination strategy. Additionally, studies reflecting a transdisciplinary approach that actively involves communities and stakeholders are crucial for effective and sustainable rabies management.

Sustainable surveillance enables timely diagnosis and efficient reporting to track outbreaks, while fostering collaboration among communities, health professionals, and policymakers for effective rabies control [1,2,50]. It provides a reliable system for assessing risks to identify high-priority areas for intervention. However, only a few studies from our review had a direct link to the surveillance system. Given that most of the studies were descriptive, this is unsurprising but also concerning, as none of the studies indicated a fully integrated or consistently applied rabies surveillance system coordinated at the district or national level. Some studies noted transitions in Uganda's surveillance systems- from paper-based Health Management Information Systems (HMIS) before 2011 to DHIS2 and mTrac for data collection afterwards [25]. Another study reported the importance of utilising passive surveillance data, although it didn't directly have a link to the surveillance system [24]. Another study examined the consistency of dog rabies case reports submitted by Southern and Eastern African countries, including Uganda, to multinational organisations, highlighting the discrepancies in reported data, emphasising the need for improved rabies surveillance and reporting systems [19]. The reliance on regional or global data instead of country-specific information may introduce further inaccuracies, as these sources may not fully represent Uganda's context [10]. Thus, the lack of an integrated surveillance system demonstrates the need for more robust rabies surveillance frameworks in Uganda, especially given the country's commitment to the Zero by 30 goal. While both human and animal surveillance systems are operational to some extent, the lack of integration between these sectors makes it difficult to accurately assess the true burden of rabies. This emphasises the need for an integrated surveillance framework, i.e. Integrated Bite Case Management, to strengthen coordination and improve rabies surveillance [51].

Despite the growing interest in rabies research in Uganda, there are only a few studies on diagnostic approaches, molecular epidemiology and genetic characterisation of the rabies virus. Two studies have addressed molecular epidemiology [28,34], leaving critical gaps in the virus's evolution, transmission dynamics and regional strain variation research, which would consequently help to inform prevention strategies. Moreover, human laboratory diagnostic facilities are still lacking, and rabies in humans is rarely confirmed through laboratory testing. For animal diagnostics, laboratories are highly centralised, with only one national laboratory, NAADEC, and one Central Diagnostic Laboratory (CDL) at COVAB, Makerere University, being able to conduct rabies diagnosis. Limited human and logistical resources for sample collection and testing further hinder surveillance, as rabies requires specialised procedures, personal protective equipment (PPE), and trained personnel. To address the challenges, strengthening regional diagnostic capacities and studies to expand the use of rapid diagnostic tests is essential. Thus, strengthening diagnostic capacities at regional levels and studies focusing on the use of rapid diagnostic tests are needed. In the absence of advanced laboratory technologies, rabies rapid diagnostic tests offer a reliable field alternative. Léchenne et al., 2016 highlight their usability and accuracy, making them valuable for rabies surveillance in resource-limited settings in countries like Chad [52]. Strengthening in-country laboratory facilities remains a critical priority to enhance rabies surveillance and diagnostics, supporting increased research efforts in this field.

In our review, one study addressed but did not conduct an economic assessment; instead, it considered social and economic aspects among five criteria for multisectoral zoonotic disease prioritisation [10]. A significant gap lies in economic research on rabies, emphasising the need for further studies on its financial burden and the cost-effectiveness of control measures. Conducting economic assessments of rabies control and prevention programs, such as mass dog vaccination campaigns, rabies surveillance programs, or human vaccination, would generate valuable evidence to inform policy recommendations [[53], [54], [55]], incorporating context-specific cost-effectiveness measures relevant to the Ugandan setting. FAIR principles were also applied in four studies. This lack of adherence raises concerns about scientific rigour, as limited data accessibility and transparency compromise the reproducibility and reliability of the research.

Our review was conducted following the PRISMA guidelines, with data assessed and screened by at least two reviewers. The PRSIMA-ScR checklist is provided in Supplementary File 3. However, this review included only those research papers that were retrieved through the systematic search. Studies that were not indexed in the databases may have been missed. Additionally, we did not conduct a deeper search of the grey literature. While we used a structured search strategy, some relevant papers may have been overlooked due to variations in terminology. Furthermore, some studies (for example, https://www.sciencedirect.com/science/article/abs/pii/S1558787824000947) were not included in our review, as they were published after our literature search was conducted in October 2024. Additionally, reviewers categorised and interpreted the information, which may have resulted in some simplification of contexts.

## 5 Conclusion

This scoping review identified 26 studies on rabies research in Uganda, highlighting critical gaps, particularly in research initiatives. Most studies conducted are largely descriptive, emphasising the need for more interventional and applied research. Future rabies studies in Uganda should focus on strengthening One Health collaborations to enhance integrated surveillance frameworks and diagnostic capacities, which are essential to achieving the ‘Zero-by-30’ commitment. Such research will provide a foundation for evidence-based policy formulation and implementation. Additionally, strengthening in-country collaborations among Ugandan researchers, experts working with sector ministries can not only build research capacity but also drive sustainable rabies control efforts.

## CRediT authorship contribution statement

**Rabina Ghimire:** Writing – review & editing, Writing – original draft, Visualization, Validation, Software, Resources, Project administration, Methodology, Investigation, Formal analysis, Data curation, Conceptualization. **Terence Odoch:** Writing – review & editing, Validation, Supervision, Investigation, Funding acquisition, Data curation, Conceptualization. **Samuel George Okech:** Writing – review & editing, Validation, Investigation, Data curation, Conceptualization. **Rose Lesley Ninsiima:** Writing – review & editing, Investigation, Data curation. **Felister Apio:** Writing – review & editing, Investigation, Data curation. **Siya Aggrey:** Writing – review & editing, Investigation, Data curation. **Felix Opiyo Lakor:** Writing – review & editing, Investigation, Data curation. **Clovice Kankya:** Writing – review & editing, Validation, Supervision, Funding acquisition, Conceptualization. **Salome Dürr:** Writing – review & editing, Validation, Supervision, Methodology, Investigation, Funding acquisition, Data curation, Conceptualization. **Sonja Hartnack:** Writing – review & editing, Validation, Supervision, Resources, Project administration, Methodology, Investigation, Funding acquisition, Formal analysis, Data curation, Conceptualization.

## Funding information

This work was funded by the 10.13039/501100001711Swiss National Science Foundation (SNSF) under the eRabies Surveillance Project – A Way Forward to Rabies Elimination in Uganda (IZSTZ0_208430).

## Declaration of competing interest

The authors declare that they have no known competing financial interests or personal relationships that could have appeared to influence the work reported in this paper.

## Data Availability

The authors declare that all the data supporting the study are available within the manuscript and its supplementary materials.

## Appendix A Supplementary data

Supplementary material 1 Image 1

Supplementary material 2 Image 2

Supplementary material 3 Image 3

## References

[1] Hampson K., Coudeville L., Lembo T., Sambo M., Kieffer A., Attlan M., Barrat J., Blanton J.D., Briggs D.J., Cleaveland S., Costa P., Freuling C.M., Hiby E., Knopf L., Leanes F., Meslin F.X., Metlin A., Miranda M.E., Müller T., Nel L.H., Recuenco S., Rupprecht C.E., Schumacher C., Taylor L., Vigilato M.A.N., Zinsstag J., Dushoffy J. (2015). Estimating the global burden of endemic canine rabies. PLoS Negl. Trop. Dis..

[2] World Health Organization (2018). https://www.who.int/publications/i/item/WHO-TRS-1012.

[3] Banyard A.C., Horton D.L., Freuling C., Müller T., Fooks A.R. (2013). Control and prevention of canine rabies: the need for building laboratory-based surveillance capacity. Antivir. Res..

[4] Sambo M., Hampson K., Johnson P.C.D., Johnson O.O. (2014). Understanding and overcoming geographical barriers for scaling up dog vaccinations against rabies. Sci. Rep..

[5] Adisasmito W.B., Almuhairi S., Behravesh C.B., Bilivogui P., Bukachi S.A., Casas N., Zhou L. (2022). One health: a new definition for a sustainable and healthy future. PLoS Pathog..

[6] FAO, UNEP, WHO, WOAH (2022).

[7] Wallace R.M., Mehal J., Nakazawa Y., Recuenco S., Bakamutumaho B., Osinubi M., Tugumizemu V., Blanton J.D., Gilbert A., Wamala J. (2017). The impact of poverty on dog ownership and access to canine rabies vaccination: results from a knowledge, attitudes and practices survey, Uganda 2013. Infect. Dis. Pov..

[8] Uganda National Institute of Public Health (30 December 2021). Dying Rabid: Adopting Compulsory Mass Dog Vaccination to Reduce Human Deaths from Dog Rabies in Uganda: Policy brief. https://uniph.go.ug/dying-rabid-adopting-compulsory-mass-dog-vaccination-to-reduce-human-deaths-from-dog-rabies-in-uganda-policy-brief/.

[9] Monje F., Kadobera D., Ndumu D.B., Bulage L., Ario A.R. (2021). Trends and spatial distribution of animal bites and vaccination status among victims and the animal population, Uganda: a veterinary surveillance system analysis, 2013–2017. PLoS Negl. Trop. Dis..

[10] Sekamatte M., Krishnasamy V., Bulage L., Kihembo C., Nantima N., Monje F., Behravesh C.B., Multisectoral prioritization of zoonotic diseases in Uganda (2017). A one health perspective. PLoS One.

[11] Iyadi L. (2022). https://rr-africa.woah.org/en/news/uganda-validates-a-national-strategy-on-rabies-elimination/.

[12] World Organisation for Animal Health (WOAH) (2018). Zero by 30: The Global Strategic Plan to End Human Deaths From Dog-Mediated Rabies by 2030. https://www.woah.org/en/document/zero_by_30_final_130618/.

[13] Global Alliance for Rabies Control (2025). https://rabiesalliance.org/country/uganda.

[14] Arksey H., O'Malley L. (2005). Scoping studies: towards a methodological framework. Int. J. Soc. Res. Methodol..

[15] Tricco A.C., Lillie E., Zarin W., O'Brien K.K., Colquhoun H., Levac D., Straus S.E. (2018). PRISMA extension for scoping reviews (PRISMA-ScR): checklist and explanation. Ann. Intern. Med..

[16] Ghimire R. (2024). A Scoping Review of Rabies Research in Uganda 2024, Protocol, OSF. https://osf.io/wu5pj.

[17] Rayyan (2024). https://rayyan.ai/.

[18] Iannone R., Roy O. (2024). https://cran.r-project.org/web/packages/DiagrammeR/index.html.

[19] Beyene T.J., Mourits M.C.M., Hogeveen H. (2017). Dog rabies data reported to multinational organizations from southern and eastern African countries. BMC. Res. Notes.

[20] Gourlaouen M., Angot A., Mancin M., Bebay C., Soumaré B., Ellero F., Benedictis P.D. (2020). An inter-laboratory trial as a tool to increase rabies diagnostic capabilities of sub-Saharan African veterinary laboratories. PLOS Neg. Trop. Dis..

[21] Okello A., Welburn S., Smith J. (2015). Crossing institutional boundaries: mapping the policy process for improved control of endemic and neglected zoonoses in sub-Saharan Africa. Health Policy Plan..

[22] Warembourg C., Wera E., Odoch T., Bulu P.M., Berger-González M., Alvarez D., Dürr S. (2021). Comparative study of free-roaming domestic dog management and roaming behavior across four countries: Chad, Guatemala, Indonesia, and Uganda. Front. Vet. Sci..

[23] Warembourg C., Fournié G., Abakar M.F., Alvarez D., Berger-González M., Odoch T., Dürr S. (2021). Predictors of free-roaming domestic dogs' contact network centrality and their relevance for rabies control. Sci. Rep..

[24] Byaruhanga J., Tayebwa D.S., Eneku W., Afayoa M., Mutebi F., Ndyanabo S., Vudriko P. (2017). Retrospective study on cattle and poultry diseases in Uganda. Int. J. Vet. Sci. Med..

[25] Masiira B., Makumbi I., Matovu J.K.B., Ario A.R., Nabukenya I., Kihembo C., Mbonye A. (2018). Long-term trends and spatial distribution of animal bite injuries and deaths due to human rabies infection in Uganda, 2001-2015. PLoS One.

[26] Wangoda R., Nakibuuka J., Nyangoma E., Kizito S., Angida T. (2019). Animal bite injuries in the accident and emergency unit at Mulago Hospital in Kampala, Uganda. Pan Afr. Med. J..

[27] Monje F., Erume J., Mwiine F.N., Kazoora H., Okech S.G. (2020). Knowledge, attitude and practices about rabies management among human and animal health professionals in Mbale District, Uganda. One Health Outlook.

[28] Hirano S., Itou T., Shibuya H., Kashiwazaki Y., Sakai T. (2010). Molecular epidemiology of rabies virus isolates in Uganda. Virus Res..

[29] Bonaparte S.C., Adams L., Bakamutumaho B., Costa G.B., Cleaton J.M., Gilbert A.T., Wallace R.M. (2021). Rabies post-exposure healthcare-seeking behaviors and perceptions: results from a knowledge, attitudes, and practices survey, Uganda, 2013. PLoS One.

[30] Kisaka S., Makumbi F.E., Majalija S., Kagaha A., Thumbi S.M. (2021). “As long as the patient tells you it was a dog that bit him, why do you need to know more?” a qualitative study of how healthcare workers apply clinical guidelines to treat dog bite injuries in selected hospitals in Uganda. PLoS One.

[31] Kisaka S., Makumbi F.E., Majalija S., Muwanga M., Thumbi S.M. (2020). Epidemiology and preclinical management of dog bites among humans in Wakiso and Kampala districts, Uganda: implications for prevention of dog bites and rabies. PLoS One.

[32] Kisaka S., Makumbi F.E., Majalija S., Bahizi G., Thumbi S.M. (2022). The potential for the double risk of rabies and antimicrobial resistance in a high rabies-endemic setting: detection of antibiotic resistance in bacterial isolates from infected dog bite wounds in Uganda. Antimicrob. Resist. Infect. Control.

[33] Kisaka S., Makumbi F., Majalija S., Bahizi G., Thumbi S.M. (2021). Delays in initiating rabies post-exposure prophylaxis among dog bite victims in Wakiso and Kampala districts, Uganda. AAS Open Res..

[34] Omodo M., Ar Gouilh M., Mwiine F.N., Okurut A.R.A., Nantima N., Namatovu A., Sekamatte M. (2020). Rabies in Uganda: rabies knowledge, attitude and practice and molecular characterization of circulating virus strains. BMC Infect. Dis..

[35] Evans M.J., Burdon Bailey J.L., Lohr F.E., Opira W., Migadde M., Gibson A.D., Mazeri S. (2019). High coverage mass rabies vaccination in rural Uganda using predominantly static point methodology. Vet. J..

[36] Fèvre E.M., Kaboyo R.W., Persson V., Edelsten M., Coleman P.G., Cleaveland S. (2005). The epidemiology of animal bite injuries in Uganda and projections of the burden of rabies. Trop. Med. Int. Health.

[37] Alobo G., Kahunde A., Luyckx V., Okech S., Semakula J., Agaba D., Hartnack S. (2020). Culling dogs to control rabies in Uganda – an example of moral distress for a veterinary officer. Berl. Münch. Tierärztl. Wochenschr..

[38] Kankya C., Dürr S., Hartnack S., Warembourg C., Okello J., Muleme J., Okello W., Methodius T., Alobo G., Odoch T. (2022). Awareness, knowledge, and perceptions regarding rabies prevention among rural communities in Masaka District, Central Uganda: a qualitative study. Front. Vet. Sci..

[39] Millán A.D., Chirife G., Kalema-Zikusoka O., Cabezón J., Muro I. Marco, Mugisha L. (2013). Serosurvey of dogs for human, livestock, and wildlife pathogens, Uganda. Emerg. Infect. Dis..

[40] Atuheire C.G.K., Okwee-Acai J., Taremwa M., Terence O., Ssali S.N., Mwiine F.N., Munyeme M., Tryland M. (2024). Descriptive analyses of knowledge, attitudes, and practices regarding rabies transmission and prevention in rural communities near wildlife reserves. Trop. Med. Health.

[41] Atuheire C.G.K., Okwee-Acai J., Taremwa M., Ssajjakambwe P., Munyeme M., Kankya C., Tryland M. (2024). Households neighboring wildlife protected areas may be at a higher risk of rabies than those located further away: a community-based cross-sectional cohort study at pian Upe game reserve, Bukedea district, eastern Uganda. Front. Trop. Dis..

[42] Pieracci E.G., Scott T.P., Coetzer A., Athman M., Mutembei A., Kidane A.H., Blanton J. (2017). The formation of the eastern Africa rabies network: a sub-regional approach to rabies elimination. Trop. Med. Infect. Dis..

[43] Nantima N., Ilukor J., Kaboyo W., Ademun A.R.O., Muwanguzi D., Sekamatte M., Bwire G. (2019). The importance of a one health approach for prioritising zoonotic diseases to focus on capacity-building efforts in Uganda. Rev. Sci. Tech. Off. Int. Epizoot..

[44] Buregyeya E., Atusingwize E., Nsamba P., Musoke D., Naigaga I., Kabasa J.D., Bazeyo W. (2020). Operationalizing the one health approach in Uganda: challenges and opportunities. Epidemiol. Glob. Health.

[45] Gelgie A.E., Cavalerie L., Kaba M., Asrat D., Mor S.M. (2022). Rabies research in Ethiopia: a systematic review. One Health.

[46] Kemunto N., Mogoa E., Osoro E., Bitek A., Njenga M.K., Thumbi S.M. (2018). Zoonotic disease research in East Africa. BMC Infect. Dis..

[47] Kanda K., Obayashi Y., Jayasinghe A., De G.S.P., Gunawardena S., Delpitiya N.Y., Priyadarshani N.G.W., Tamashiro H. (2015). Outcomes of a school-based intervention on rabies prevention among school children in rural Sri Lanka. Int. Health.

[48] Barroga T.R.M., Basitan I.S., Lobete T.M., Bernales R.P., Gordoncillo M.J.N., Lopez E.L., Abila R.C. (2018). Community awareness on rabies prevention and control in Bicol, Philippines: pre- and post-project implementation. Trop. Med. Infect. Dis..

[49] Neevel A.M.G., Hemrika T., Claassen E., van de Burgwal L.H.M. (2018). A research agenda to reinforce rabies control: a qualitative and quantitative prioritization. PLoS Negl. Trop. Dis..

[50] Rupprecht C.E., Kuzmin I.V., Yale G., Nagarajan T., Meslin F.-X. (2019). Priorities in applied research to ensure programmatic success in the global elimination of canine rabies. Vaccine.

[51] Léchenne M., Traore A., Hattendorf J., Kallo V., Oussiguere A., Tetchi M., Zinsstag J. (2021). Increasing rabies data availability: the example of a one health research project in Chad, Côte d'Ivoire and Mali. Acta Trop..

[52] Léchenne M., Naïssengar K., Lepelletier A., Alfaroukh I.O., Bourhy H., Zinsstag J., Dacheux L. (2016). Validation of a rapid rabies diagnostic tool for field surveillance in developing countries. PLoS Negl. Trop. Dis..

[53] Borse R.H., Atkins C.Y., Gambhir M., Undurraga E.A., Blanton J.D., Kahn E.B., Meltzer M.I. (2018). Cost-effectiveness of dog rabies vaccination programs in East Africa. PLoS Negl. Trop. Dis..

[54] Taylor E., Prada J.M., Vilas V.D.R., Undurraga E.A., Wallace R., Horton D.L. (2023). Cost-effectiveness analysis of integrated bite case management and sustained dog vaccination for rabies control. Am. J. Trop. Med. Hyg..

[55] Ritchie A.J., Meeyai A., Trotter C., Douglas A.D. (2025). Routine childhood rabies pre-exposure prophylaxis can be cost effective in low- and middle-income countries. Vaccine.

