# Supplementary material for: Rabies research in Uganda – A scoping review
Source: One Health. 2025 Oct 14;21:101240. doi: 10.1016/j.onehlt.2025.101240 (PMC12555810; doi:10.1016/j.onehlt.2025.101240)
Supplement: Supplementary file 1 — Supplementary material 1 [file mmc1.docx]

**Annex 1**

Search strategy for literature search in PubMed

((Rabies[mh] OR Rabies Vaccines[mh] OR Rabies Virus[mh] OR lyssavirus[mh] OR rabies OR rabid OR rabies-burden OR "rabies burden" OR "rabies vaccination" OR lyssavirus) AND (dogs[mh] OR cats[mh] OR Animals, Wild[mh] OR goats[mh] OR cattle[mh] OR dog OR dogs OR canine OR "dog bites" OR cat OR cats OR wildlife OR "wild animal" OR goat OR goats OR bovine OR bovines OR cattle OR cattles OR "animal bite" OR "animal bite injuries" OR "animal bite injury")) AND (Uganda[mh] OR Africa, Eastern[Mesh:NoExp] OR Africa South of the Sahara[Mesh:NoExp] OR uganda OR ugandan OR "east africa" OR "eastern africa" OR "sub sahara" OR "Sub-Saharan Africa" OR "Subsaharan Africa")
