# Supplementary material for: Rabies research in Uganda – A scoping review
Source: One Health. 2025 Oct 14;21:101240. doi: 10.1016/j.onehlt.2025.101240 (PMC12555810; doi:10.1016/j.onehlt.2025.101240)
Supplement: Supplementary file 2 — Supplementary material 2 [file mmc2.docx]

Supplementary File 2

Table of all included publications with their reference number and doi.

| Study | Date of publication | Authors | Reference | Doi |
| --- | --- | --- | --- | --- |
| The epidemiology of animal bite injuries in Uganda and projections of the burden of rabies | 2005 | Fèvre, E. M.; Kaboyo, R. W.; Persson, V.; Edelsten, M.; Coleman, P. G.; Cleaveland, S. | [36] | DOI: 10.1111/j.1365-3156.2005.01447.x |
| Molecular epidemiology of rabies virus isolates in Uganda | 2010 | Hirano, S.; Itou, T.; Shibuya, H.; Kashiwazaki, Y.; Sakai, T.; | [28] | DOI: 10.1016/j.virusres.2009.10.003 |
| Serosurvey of dogs for human, livestock, and wildlife pathogens, Uganda | 2013 | Millán, J.; Chirife, A. D.; Kalema-Zikusoka, G.; Cabezón, O.; Muro, J.; Marco, I.; Cliquet, F.; León-Vizcaíno, L.; Wasniewski, M.; Almería, S.; Mugisha, L.; | [39] | DOI: 10.3201/eid1904.121143 |
| Crossing institutional boundaries: mapping the policy process for improved control of endemic and neglected zoonoses in sub-Saharan Africa | 2015 | Okello, A.; Welburn, S.; Smith, J.; | [21] | DOI: 10.1093/heapol/czu059 |
| The impact of poverty on dog ownership and access to canine rabies vaccination: Results from a knowledge, attitudes and practices survey, Uganda 2013 | 2017 | Wallace, R. M. L.; Mehal, J.; Nakazawa, Y.; Recuenco, S.; Bakamutumaho, B.; Osinubi, M.; Tugumizemu, V.; Blanton, J. D.; Gilbert, A.; Wamala, J | [7] | DOI: 10.1186/s40249-017-0306-2 |
| Dog rabies data reported to multinational organizations from Southern and Eastern African countries | 2017 | Beyene, T. J.; Mourits, M. C. M.; Hogeveen, H.; | [19] | DOI: 10.1186/s13104-017-2527-7 |
| Retrospective study on cattle and poultry diseases in Uganda | 2017 | Byaruhanga, J.; Tayebwa, D. S.; Eneku, W.; Afayoa, M.; Mutebi, F.; Ndyanabo, S.; Kakooza, S.; Okwee-Acai, J.; Tweyongyere, R.; Wampande, E. M.; Vudriko, P.; | [24] | [DOI: 10.1016/j.ijvsm.2017.07.001](https://doi.org/10.1016/j.ijvsm.2017.07.001) |
| Multisectoral prioritization of zoonotic diseases in Uganda, 2017: A One Health perspective | 2018 | Sekamatte, M.; Krishnasamy, V.; Bulage, L.; Kihembo, C.; Nantima, N.; Monje, F.; Ndumu, D.; Sentumbwe, J.; Mbolanyi, B.; Aruho, R.; Kaboyo, W.; Mutonga, D.; Basler, C.; Paige, S.; Barton Behravesh, C. | [10] | DOI: 10.1371/journal.pone.0196799 |
| Long term trends and spatial distribution of animal bite injuries and deaths due to human rabies infection in Uganda, 2001-2015 | 2018 | Masiira, B.; Makumbi, I.; Matovu, J. K. B.; Ario, A. R.; Nabukenya, I.; Kihembo, C.; Kaharuza, F.; Musenero, M.; Mbonye, A.; | [25] | DOI: 10.1371/journal.pone.0198568 |
| Animal bite injuries in the accident and emergency unit at Mulago hospital in Kampala, Uganda | 2019 | Wangoda, R.; Nakibuuka, J.; Nyangoma, E.; Kizito, S.; Angida, T. | [26] | DOI: 10.11604/pamj.2019.33.112.16624 |
| Implementation of high coverage mass rabies vaccination in rural Uganda using predominantly static point methodology | 2019 | Evans, M. J.; Burdon Bailey, J. L.; Lohr, F. E.; Opira, W.; Migadde, M.; Gibson, A. D.; Handel, I. G.; Bronsvoort, B. M. D.; Mellanby, R. J.; Gamble, L.; Mazeri, S.; | [35] | DOI: 10.1016/j.tvjl.2019.04.013 |
| An inter-laboratory trial as a tool to increase rabies diagnostic capabilities of sub-saharan african veterinary laboratories | 2020 | Gourlaouen, M.; Angot, A.; Mancin, M.; Bebay, C.; Soumaré, B.; Ellero, F.; Zecchin, B.; Leopardi, S.; De Battisti, C.; Terregino, C.; de Benedictis, P | [20] | DOI: 10.1371/journal.pntd.0008010 |
| Knowledge, attitude and practices about rabies management among human and animal health professionals in Mbale District, Uganda | 2020 | Monje, F.; Erume, J.; Mwiine, F.; Kazoora, H.;, Kech, S. G | [27] | [10.1186/s42522-020-00031-6](https://doi.org/10.1186/s42522-020-00031-6) |
| Epidemiology and preclinical management of dog bites among humans in Wakiso and Kampala districts, Uganda: Implications for prevention of dog bites and rabies | 2020 | Kisaka, S.; Makumbi, F. E.; Majalija, S.; Bangirana, A.; Thumbi, S. M.; | [31] | DOI: 10.1371/journal.pone.0239090 |
| Rabies in Uganda: rabies knowledge, attitude and practice and molecular, characterization of circulating virus strains | 2020 | Omodo, M.; Ar Gouilh, M.; Mwiine, F. N.; Okurut, A. R. A.; Nantima, N.; Namatovu, A.; Nakanjako, M. F.; Isingoma, E.; Arinaitwe, E.; Esau, M.; Kyazze, S.; Bahati, M.; Mayanja, F.; Bagonza, P.; Urri, R. A.; Lovincer, M. N.; Nabatta, E.; Kidega, E.; Ayebazibwe, C.; Nakanjako, G.; Sserugga, J.; Ndumu, D. B.; Mwebe, R.; Mugabi, K.; Gonzalez, J. P.; Sekamatte, M. | [34] | <https://doi.org/10.1186/s12879-020-4934-y> |
| Culling dogs to control rabies in Uganda – An example of moral distress for a veterinary officer | 2020 | Alobo, G.; Kahunde, M. A.; Luyckx, V.; Okech, S. G.; Semakula, J. R.; Agaba, D.; Hartnack, S | [37] | DOI: 10.2376/0005-9366-19052 |
| Trends and spatial distribution of animal bites and vaccination status among victims and the animal population, Uganda: A veterinary surveillance system analysis, 2013–2017 | 2021 | Monje, F.; Kadobera, D.; Ndumu, D. B.; Bulage, L.; Ario, A. R.; | [9] | DOI: 10.1371/journal.pntd.0007944 |
| Comparative Study of Free-Roaming Domestic Dog Management and Roaming Behavior Across Four Countries: Chad, Guatemala, Indonesia, and Uganda | 2021 | Warembourg, C.; Wera, E.; Odoch, T.; Bulu, P. M.; Berger-González, M.; Alvarez, D.; Abakar, M. F.; Maximiano Sousa, F.; Cunha Silva, L.; Alobo, G.; Bal, V. D.; López Hernandez, A. L.; Madaye, E.; Meo, M. S.; Naminou, A.; Roquel, P.; Hartnack, S.; Dürr, S.; | [22] | 10.3389/fvets.2021.617900 |
| Predictors of free-roaming domestic dogs' contact network centrality and their relevance for rabies control | 2021 | Warembourg, C.; Fournié, G.; Abakar, M. F.; Alvarez, D.; Berger-González, M.; Odoch, T.; Wera, E.; Alobo, G.; Carvallo, E. T. L.; Bal, V. D.; López Hernandez, A. L.; Madaye, E.; Maximiano Sousa, F.; Naminou, A.; Roquel, P.; Hartnack, S.; Zinsstag, J.; Dürr, S. | [23] | DOI: 10.1038/s41598-021-92308-7 |
| Rabies post-exposure healthcare-seeking behaviors and perceptions: Results from a knowledge, attitudes, and practices survey, Uganda, 2013 | 2021 | Bonaparte, S. C.; Adams, L.; Bakamutumaho, B.; Costa, G. B.; Cleaton, J. M.; Gilbert, A. T.; Osinubi, M.; Pieracci, E. G.; Recuenco, S.; Tugumizemu, V.; Wamala, J.; Wallace, R. M.; | [29] | [DOI: 10.1371/journal.pone.0251702](https://doi.org/10.1371/journal.pone.0251702) |
| “As long as the patient tells you it was a dog that bit him, why do you need to know more?” A qualitative study of how healthcare workers apply clinical guidelines to treat dog bite injuries in selected hospitals in Uganda | 2021 | Kisaka, S.; Makumbi, F. E.; Majalija, S.; Kagaha, A.; Thumbi, S. M.; | [30] | DOI: 10.1371/journal.pone.0254650 |
| Delays in initiating rabies post-exposure prophylaxis among dog bite victims in Wakiso and Kampala districts, Uganda | 2021 | Kisaka, S.; Makumbi, F.; Majalija, S.; Bahizi, G.; Thumbi, S. M.; | [33] | DOI: 10.12688/aasopenres.13311.3 |
| The potential for the double risk of rabies and antimicrobial resistance in a high rabies endemic setting: detection of antibiotic resistance in bacterial isolates from infected dog bite wounds in Uganda. | 2022 | Kisaka S; Makumbi FE; Majalija S; Muwanga M; Thumbi SM | [32] | [DOI: 10.1186/s13756-022-01181-0](https://doi.org/10.1186/s13756-022-01181-0) |
| Awareness, Knowledge, and Perceptions Regarding Rabies Prevention Among Rural Communities in Masaka District, Central Uganda: A Qualitative Study | 2022 | Kankya, C.; Dürr, S.; Hartnack, S.; Warembourg, C.; Okello, J.; Muleme, J.; Okello, W.; Methodius, T.; Alobo, G.; Odoch, T. | [38] | DOI: 10.3389/fvets.2022.863526 |
| Descriptive analyses of knowledge, attitudes, and practices regarding rabies transmission and prevention in rural communities near wildlife reserves in Uganda: a One Health cross-sectional study | 2024 | Atutheire, C. G,K., Okwee-Acai, J., Taremwa, M., Odoch, T., N. Ssali, S., Mwiine, F.N., Kankya, C., Skerve, E., Tryland, M | [40] | https://doi.org/10.1186/s41182-024-00615-2 |
| Households neighboring wildlife protected areas may be at a higher risk of rabies than those located further away: a community-based crosssectional cohort study at Pian Upe game reserve, Bukedea district, Eastern Uganda | 2024 | Atuheire C, J. Okwee-Acai, M. Taremwa, P. Ssajjakambwe, M. Munyeme, C. Kankya, T. Odoch, S.N. Ssali, F. N. Mwiine, K. J. Buhler, M. Tryland | [41] | DOI 10.3389/fitd.2024.1272141 |
